# The Effects of Ankle Versus Plantar Vibrotactile Orthoses on Joint Position Sense and Postural Control in Individuals with Functional Ankle Instability: A Pilot Randomized Trial

**DOI:** 10.3390/bioengineering13020138

**Published:** 2026-01-25

**Authors:** Hanieh Khaliliyan, Mahmood Bahramizadeh, Ebrahim Sadeghi-Demneh

**Affiliations:** 1 Musculoskeletal Research Center, School of Rehabilitation Sciences, Isfahan University of Medical Sciences, Isfahan 8174673461, Iran; haniehkhaliliyan@yahoo.com; 2 Neuromusculoskeletal Rehabilitation Research Center, University of Social Welfare and Rehabilitation Sciences, Tehran 1985713834, Iran

**Keywords:** ankle instability, focal vibration, orthoses, position sense, balance, ankle sprain, proprioception

## Abstract

Functional ankle instability (FAI) is a common consequence of lateral ankle sprains, characterized by impaired sensorimotor control. While orthoses and localized vibration have shown individual benefits for FAI, their combined application in a wearable device has not been previously investigated. This pilot randomized trial compared the effects of a vibrotactile foot orthosis (VFO) and a vibrotactile ankle orthosis (VAO) on joint position sense (JPS) and postural control in individuals with FAI. Sixteen participants were randomized to receive either a VFO or a VAO, both delivering 30–50 Hz pulsed vibration in 20 min sessions, three times a week, for two weeks. Outcome measures included joint position sense (JPS) error (°), center of pressure (COP) velocity (mm/s), the Star Excursion Balance Test (SEBT), and the Six-Meter Hop Test (SMHT), which were assessed pre-intervention, immediately post-intervention, and after two weeks of use. The analysis showed a statistically significant interaction between time and intervention group for JPS error (*p* = 0.02, η^2^ = 0.42). Specifically, the VFO group improved JPS significantly more than VAO at two weeks follow-up (MD = −1.75°, *p* = 0.005, d = −1.68). Both groups significantly reduced in anteroposterior COP velocity after two weeks (VFO: MD = 1, *p* = 0.003, d = 1.47; VAO: MD = 1.39, *p* ˂ 0.001, d = 2.05) with no between-group differences. No changes were observed in the SEBT or SMHT. Plantar-based vibrotactile stimulation was more effective than ankle-based stimulation in enhancing proprioceptive acuity in individuals with FAI. Both interventions improved static postural stability, supporting the potential of integrated vibrotactile orthoses in FAI rehabilitation. No major practical issues were reported during the intervention. Two participants experienced minor discomfort related to the electronic housing bulk in the first week, which was resolved by week two. No further complaints regarding device weight or usability were observed.

## 1. Introduction

The ankle joint bears full body weight during standing and serves as the focal point for dynamic stress vectors during walking and running [1]. This joint is prone to ligamentous injury because it bears the greatest force per unit area of any human synovial joint [2]. Foot and ankle injuries are common in physically active populations [3,4,5], with lateral ankle sprains (LAS) accounting for over 70% of ankle injuries [5] and 16.2% of all injuries reported among U.S. collegiate athletes [6]. Some studies report a significant association between a history of LAS and an increased risk of knee injury [2,3]. Additionally, repetitive sprains may contribute to the development of osteoarthritis and ankylosis [7].

Between 45% and 70% of acute LAS cases involve mechanical trauma that compromises the sensorimotor control loop by upsetting the anterior talofibular and calcaneofibular ligaments’ afferent sensory architecture [8]. Functional ankle instability (FAI) may arise following a LAS, characterized by repeated LASs and a lasting sense of instability despite the absence of mechanical laxity, persisting for over a year [6,7]. FAI refers to the subjective sensation of ankle “giving way” and deficits in neuromuscular control following LAS, without necessarily involving mechanical laxity [6]. Chronic Ankle Instability (CAI), as more consistently defined in the recent literature, encompasses both mechanical and functional instability [5]. In this study, we use FAI to denote the functional component of CAI, focusing on proprioceptive and balance impairments, while excluding individuals with mechanical instability [7]. FAI arises from the disruption of the afferent neural pathways in the lateral ankle joint ligaments [9]. Following an acute LAS, partial deafferentation of joint mechanoreceptors may occur, resulting in diminished proprioceptive acuity and impaired joint position sense (JPS) [8]. The sensory deficits of LAS disrupt reflexive neuromuscular control, impairing static and dynamic postural control [10], delaying peroneal reflex latency [11], inducing muscle inhibition [12], and altering weight-bearing kinematics and kinetics [13].

Foot and ankle proprioception is crucial for JPS [14]. Cutaneous mechanoreceptors detect ground reaction force (GRF), influencing muscle tone via gamma motor neurons [14,15]. Muscle spindles, activated by postural disturbances, transmit proprioceptive signals to the central nervous system (CNS) [15]. Simultaneously, plantar and ankle tactile cues inform the CNS about ankle torque, weight transfer, and limb loading velocity [16]. Integration of these sensory inputs within the CNS fine-tunes alpha motor neuron output, improving postural stability [15,16]. Human cutaneous tissue is highly sensitive to mechanical vibration, which modulates neural signal transmission both peripherally and centrally. Vibrotactile stimulation activates Meissner’s corpuscles (involved in reactive postural adjustments) and Pacinian corpuscles (involved in anticipatory control) [14]. At the molecular level, vibratory stimuli can induce hydrogen peroxide formation in biological fluids [17], a redox signaling molecule that may influence mechano-transduction pathways [18].

Previous studies have highlighted the role of lateral ankle cutaneous feedback in regulating eversion movements, as mechanoreceptors in this region detect inversion-eversion changes and transmit them to the CNS [9]. In FAI, collagen fibers of the lateral ankle skin remain structurally intact, but mechanoreceptors are more susceptible to injury during sprains due to their lower tensile tolerance compared to connective tissue [8]. This damage contributes to impaired afferent input. Also, the plantar surface of the foot contains a high density of mechanoreceptors that provide proprioceptive information related to touch, pressure, and joint movement [15,16]. It is not yet clear whether vibrotactile stimulation applied at the ankle or at the plantar surface is more effective in improving joint position sense and postural control in individuals with FAI.

Wearable biomechanical devices, such as foot orthoses (FOs) and ankle orthoses (AOs), can influence muscle strength [19], joint kinetics and kinematics [20], plantar pressure [21], functional performance [22], and postural control [23]. FOs and AOs are utilized by individuals with FAI to prevent LASs by modifying the biomechanics of the subtalar and talocrural joints, respectively [19,22]. People with FAI often display altered biomechanics due to sensory deficits, which may include increased foot inversion, plantarflexion relative to the tibia, and lateral displacement of the center of pressure (COP) during walking [13]. Heel cup FOs are frequently prescribed to correct the lateral deviation of the COP by positioning the subtalar joint in a neutral position for individuals with FAI [21]. When combined with medial arch support and a metatarsal pad, the heel cup provides more effective plantar pressure redistribution than when used alone [20].

AOs are prescribed for individuals with FAI to stabilize the talocrural joint and are classified as soft types, like neoprene sleeves, or semi-rigid types, such as stirrup or double-upright orthoses. While semi-rigid orthoses provide greater mechanical stability, they may limit frontal-plane motion, potentially impairing shock absorption and adaptive postural responses during dynamic tasks [23,24]. A restricted range of motion diminishes the capacity to dissipate GRFs through optimal joint kinematics [13,25]. In this context, soft AOs are a preferable option due to their biomechanical properties, which offer targeted compression and flexibility while preserving the ankle’s normal range of motion.

Although prior studies have shown that localized vibration stimulation [16] or orthoses [19,20,21,22,23] can independently improve sensorimotor function, each typically targets only one aspect of the sensorimotor loop, either sensory-perceptual input or motor-behavioral output, in isolation. To our knowledge, no study has integrated vibrotactile stimulation with orthotic support to concurrently address both afferent (proprioceptive) deficits and biomechanical instability in individuals with FAI. Grounded in the principle that sensory input directly shapes motor output, our dual-modality intervention combines these approaches to synergistically enhance proprioceptive accuracy and neuromuscular control.

The objective of this pilot study was to compare the immediate and short-term (two-week) effects of vibrotactile stimulation delivered through wearable devices, vibrotactile ankle orthoses (VAO), and vibrotactile foot orthoses (VFO), at two distinct anatomical sites (ankle versus plantar surface) on proprioceptive acuity and postural control in individuals with FAI.

## 2. Materials and Methods

### 2.1. Trial Design, Setting, and Registration

This study was a two-arm, parallel-group, repeated-measures pilot randomized trial with a 1:1 allocation ratio. The protocol was approved by the Ethics Committee of Isfahan University of Medical Sciences (ID: IR.MUI.NUREMA.REC.1403.029). The trial was prospectively registered in the Iranian Registry of Clinical Trials (IRCT20240514061793N1; https://irct.behdasht.gov.ir/trial/76942 (accessed on 7 November 2025)). It was reported in accordance with the CONSORT 2025 statement [26]. All assessments were conducted at the Musculoskeletal Research Center, Isfahan University of Medical Sciences, Isfahan, Iran. The registered protocol was not modified after study initiation. Participants were recruited through outpatient clinics and sports facilities associated with Isfahan University of Medical Sciences.

### 2.2. Eligibility Criteria

Inclusion criteria were based on the Ankle Consortium criteria for FAI. Eligible individuals were required to: (1) be aged between 18 and 35; (2) self-report recurrent LASs; (3) experienced at least two episodes of subjective ankle instability during the preceding six months; (4) had experienced at least one clinically significant LAS during the previous 12 months (defined as an acute injury involving pain, swelling, and restricted range of motion lasting at least 24 h); and (5) score below 24 on the Cumberland Ankle Instability Tool (CAIT) [27].

Foot posture was assessed using the Foot Posture Index (FPI-6), a validated clinical tool that evaluates six criteria of foot alignment and morphology. Each item is scored from −2 to +2, yielding a total score between −12 (highly supinated/pes cavus) and +12 (highly pronated/pes planus). Participants with FPI scores ≥ +6 (flat foot) or ≤−6 (high arch) were excluded. Only individuals with neutral foot posture (FPI scores between −5 and +5) were included in the study [28]. Exclusion criteria were: (1) any underlying neuromusculoskeletal disorder (e.g., idiopathic scoliosis, kyphosis, Charcot arthropathy, pes planus, or pes cavus); (2) a history of lower limb fracture, or surgery; (3) bilateral FAI; and (4) the use of any biomechanical or sensory-enhancing rehabilitation modalities (e.g., orthoses, taping, and focal vibration) within the six weeks prior to enrollment. Participants were withdrawn if they expressed an unwillingness to continue or developed any new musculoskeletal injury that could affect JPS or postural control during the intervention period [25].

### 2.3. Interventions

**Figure 1:**
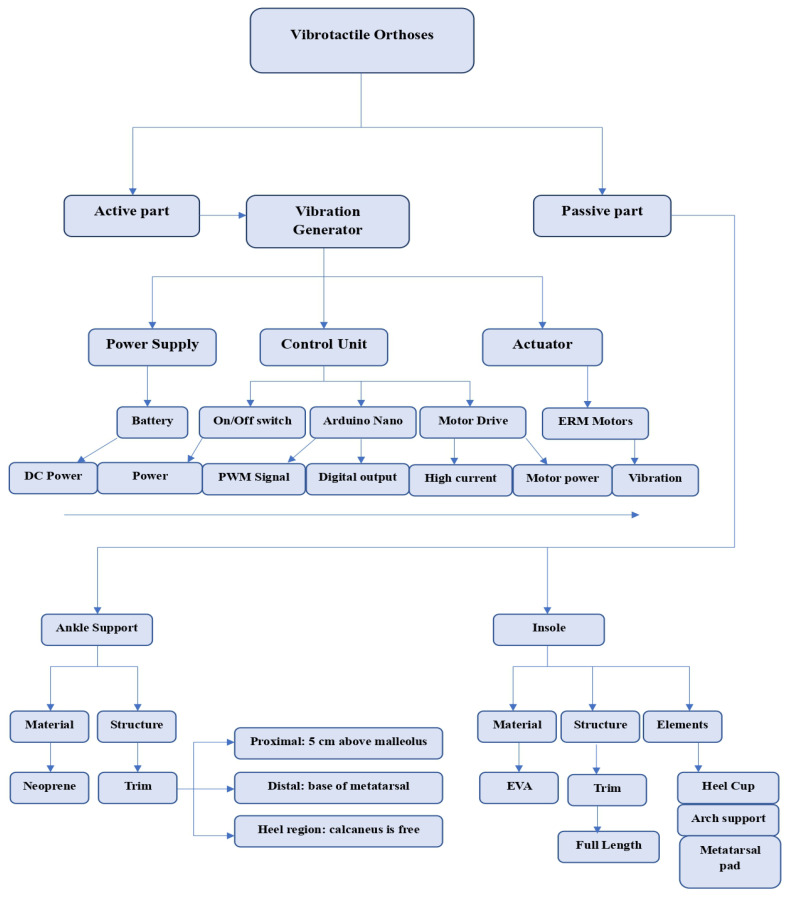
Diagram of vibrotactile orthoses.

Two configurations of the vibrotactile orthoses were developed: (1) VAO and (2) VFO. The vibration system delivers perceptible vibration in the frequency range of 30–50 Hz with an amplitude of 0.5–1 mm [29], which corresponds to the optimal sensitivity range of fast-adapting afferent nerve fibers involved in proprioception and tactile feedback [14]. Diagrams of both device configurations are presented in Figure 1.

#### Fabrication of Vibrotactile Orthoses

**Figure 2:**
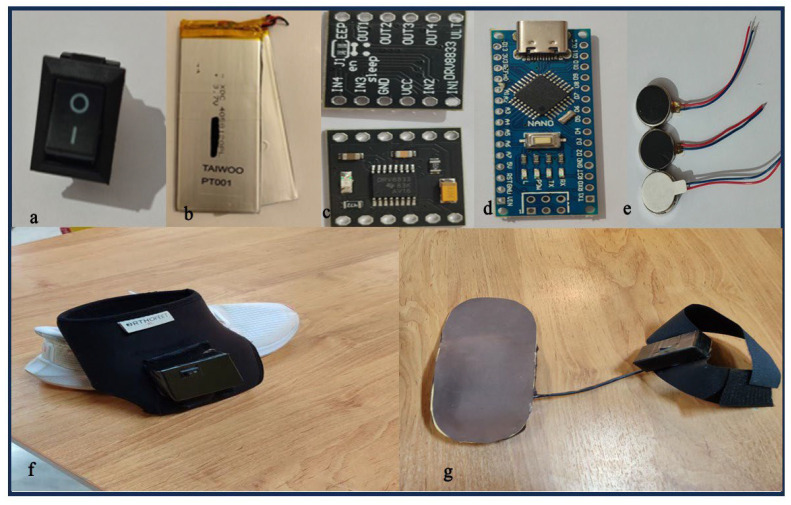
Components of the vibration system and final devices. (**a**) on/off switch; (**b**) LiPo battery; (**c**) DRV8833 motor driver modules; (**d**) Arduino Nano microcontroller; (**e**) Coin vibration motors; (**f**) vibrotactile ankle orthosis; (**g**) vibrotactile foot orthoses.

Both devices were fabricated in close collaboration between a certified orthotist and an electronics engineer. During material selection and structural design of both active and passive parts, attention was given to minimizing bulk and overall weight. The vibration system comprises the following components: a LiPo battery (2 cells, 7.4 volt, TAIWOO PT001, Seoul, Korea), an Arduino Nano (CH340 USB–Serial chip, WCH, Nanjing, China) with ATmega328P Microchip (Microchip Technology Inc., Chandler, AZ, USA), two DC motor drivers (DRV8833, Texas Instruments Inc., Dallas, TX, USA), an on/off switch, and three-coin vibration motors (9000 RPM, Jinlong Machinery & Electronics Co., Ltd., Jinjiang, China) (Figure 2a–e). The Arduino Nano CH340 is programmed in C++ using the Arduino IDE software (Version 2.0, Arduino, Ivrea, Italy) to generate Pulse-width modulation (PWM) signals that drive the DRV8833 motor driver modules. The final vibration system weight was 200 g. All units were calibrated prior to participant use with a laser vibrometer (Polytec OFV-505, Waldbronn, Germany) to ensure uniformity in vibration frequency, amplitude, and duty cycle across devices. The stimulation protocol utilized a pulsed pattern, 500 ms of vibration followed by 500 ms of rest, throughout each 20 min intervention session.

AO was provided using a prefabricated soft neoprene ankle support (ORTHOFEET Inc., Tehran, Iran) with a thickness of 0.5 cm. Sizing was based on predefined ankle circumference categories: 20–22 cm (small), 22–24 cm (medium), 24–26 cm (large), and 26–28 cm (extra-large). FO was also a prefabricated insole constructed from ethylene-vinyl acetate (EVA) foam with a Shore A50 hardness. It incorporated a 10 mm heel cup, a 15 mm medial arch support, and a 5 mm EVA metatarsal pad.

To integrate the vibration system with AOs, polyester-based Velcro was attached to the lateral aspect of the AO. The vibration motors were positioned to target mechanoreceptor-rich areas around the lateral malleolus due to initial injury to the cutaneous and ligaments of this area [8]. The backing layer of the Velcro was modified by creating circular cutouts for fixing vibration motors, corresponding to the anatomical locations of the posterior, anterior, and inferior aspects of the lateral malleolus. A thermoplastic housing box (10 × 10 × 2 cm, 15 g) was affixed to the orthosis to securely hold the power supply, control module, and power switch. The final VAO is shown in Figure 2f.

To integrate the vibration system into FOs, the insole was perforated at three locations: beneath the first metatarsal head, the fifth metatarsal head, and the center of the heel. The rationale for selecting these locations was based on consensus from prior research indicating a high concentration of mechanoreceptors in these regions [14]. Narrow channels were also created to accommodate the connecting wires. The vibration motors were securely mounted within these perforations, and the upper layer of the insole was covered with a thin (0.5 cm) EVA foam cushion to ensure comfort and conceal the embedded components. The remaining electronic components (Arduino Nano, motor drivers, battery, and switch) were housed in a lightweight thermoplastic enclosure (10 × 10 × 2 cm, 15 g), which was attached to a Velcro strap. This strap was secured around the distal lower leg, just above the malleoli, during device use. The final VFO is shown in Figure 2g.

### 2.4. Randomization and Blinding

Randomization was carried out using a computer-generated permuted-block schedule with a block size of 4, created via Randomizer.org (https://www.randomizer.org/). Allocation sequences were concealed in sequentially numbered, opaque, sealed envelopes prepared by an independent researcher who was not involved in participant recruitment or assessments. Upon enrollment and after baseline assessments, the assessor opened the next envelope to assign the participant to either one of the two groups. Due to the nature of the interventions (tangible wearable devices with perceptible vibration), participants could not be blinded to their assigned group. Also, to minimize order effects, the sequence of tests was randomized using a computer-generated permuted-block schedule created via Randomizer.org. Sequences were concealed in sequentially numbered, opaque envelopes, and the assessor opened the next envelope immediately before testing to determine the assessment order.

### 2.5. Outcomes

All assessments were conducted in a laboratory environment (temperature: ~22 °C, relative humidity: 15%, illumination: 120 lux, ambient noise: 40 dB). The primary outcomes were joint position sense (JPS) error (in degrees) and postural sway velocity, quantified as center of pressure (COP) velocity in millimeters per second (mm/s). Secondary outcomes included performance on two clinical functional tests: the Star Excursion Balance Test (SEBT), with reach distances normalized to leg length, and the Six-Meter Hop Test (SMHT), with completion time recorded in seconds (s).

#### 2.5.1. Joint Position Sense

**Figure 3:**
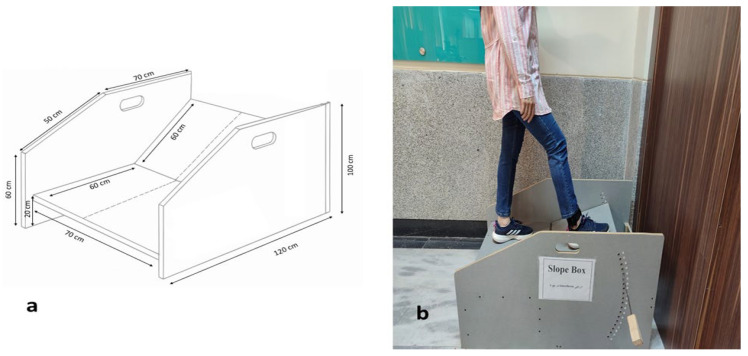
Joint position sense assessment. (**a**) Schematic illustration of the slope box; (**b**) Participants stood on the inclined platform and estimated the slope angle on a calibrated scale.

JPS was defined as the participant’s ability to discriminate slope angles while standing on an adjustable slope box (Figure 3a). This measure has demonstrated excellent test–retest reliability in individuals with FAI (ICC = 0.82) [30]. The device consisted of two main components: a fixed horizontal base and a movable inclined platform that could be adjusted in 2.5° increments. The angular range for JPS testing was set at ±22.5°, representing a physiologically relevant excursion of ankle plantarflexion/dorsiflexion that challenges proprioceptive acuity without exceeding safe limits for individuals with FAI [6]. The increment of 2.5° was selected to provide sufficient resolution for detecting subtle differences in joint position sense, consistent with thresholds reported in prior proprioception studies [31].

During testing, participants stood on the inclined platform with their affected limb, eyes closed, and head facing forward. They were instructed to estimate the slope angle on a numerical scale from −9 (corresponding to −22.5°) to +9 (corresponding to +22.5°) (Figure 3b). Before the test trials, participants underwent a familiarization phase in which they were exposed to three reference angles: −6 (−15°), 0 (neutral), and +6 (+15°) to calibrate their perceptual scale [31].

In the actual assessment, ten randomized slope angles within the full range (−22.5° to +22.5°) were presented. After each trial, the platform was returned to the neutral position to minimize the influence of muscle thixotropy on ankle proprioception. The absolute difference between the actual angle and the participant’s reported score was calculated for each trial, and the mean of these ten absolute errors was used as the primary JPS outcome measure.

#### 2.5.2. Static Postural Control

**Figure 4:**
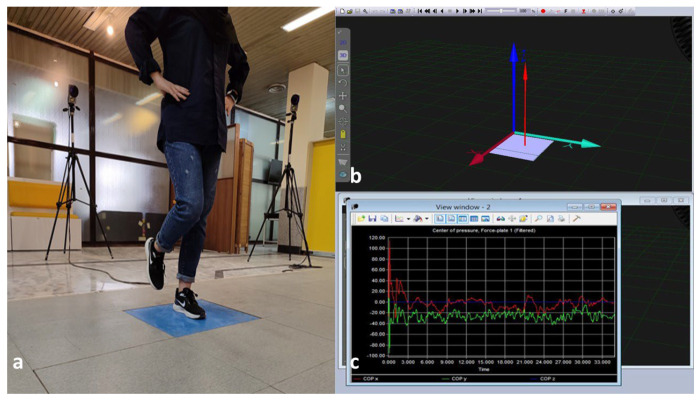
Static postural control assessment. (**a**) Single leg stance on force plate; (**b**) 3D illustration of GRF position on force plate; (**c**) COP record in the three directions.

Static postural stability was assessed using a single-leg stance task on a Kistler^®^ force plate (Model 9260AA6; dimensions: 50 × 60 cm; Kistler Instrument AG, Winterthur, Switzerland) (Figure 4a). During single-leg stance, elevated GRFs may contribute to greater knee abduction moments and increased ankle supination, both of which are biomechanical risk factors for LAS [32,33]. This protocol has demonstrated excellent test–retest reliability in individuals with FAI, with an ICC of 0.88 for COP velocity measures [34]. Participants stood on their affected limb at the center of the force plate, with foot placement standardized using a plastic film template to ensure a consistent base of support across assessments. The contralateral limb was lifted with the knee flexed to approximately 70° and the hip flexed to approximately 30°. Hands were placed bilaterally on the iliac crests, and participants fixated their gaze on a visual target positioned at eye level, 3 m ahead. The force plate featured a metal sandwich cover with four embedded 3D piezoelectric sensors recessed flush into a walkway, with the *Y*-axis aligned to the participant’s forward direction.

COP trajectories were recorded at a sampling frequency of 100 Hz over three 35 s trials. The first 5 s of each trial were discarded to eliminate transient postural adjustments, leaving a 30 s segment for analysis. The plate outputs six analog signals corresponding to three orthogonal GRF components (Figure 4b) (Fx: anterior–posterior, Fy: medio-lateral, Fz: vertical) and three moment components (Mx, My, Mz). These signals were digitized in real time using Qualisys Track Manager software (Version 2.48, Qualisys AB, Göteborg, Sweden) and subsequently filtered using a Butterworth low-pass filter with a cutoff frequency of 10 Hz (Figure 4c). The data were exported to Microsoft Excel (2019, Microsoft Corp., Redmond, WA, USA), and outcome calculations were performed. The mean of the three trials was used for statistical analysis. COP velocity is derived by computing the displacement of the COP over time, essentially the rate of change of CoP position, and has been validated in postural control studies [35]. The anteroposterior (AP) and mediolateral (ML) velocity components [Equations (1) and (2)], along with the resultant COP velocity [Equation (3)], were calculated as follows [35]. These velocity-based metrics reflect the temporal dynamics of postural sway and are considered highly sensitive to sensorimotor deficits in FAI [34]:

(1) $$Mean\;Velocity\;AP=\frac{1}{T}\sum_{i=1}^{n-1}\sqrt{{\left({AP}_{i+1}-{AP}_{i}\right)}^{2}}$$

(2) $$Mean\;Velocity\;ML=\frac{1}{T}\sum_{i=1}^{n-1}\sqrt{{\left({ML}_{i+1}-{ML}_{i}\right)}^{2}}$$

(3) $$Mean\;Velocity\;Resultant=\frac{1}{T}\sum_{i=1}^{n-1}\sqrt{{\left({AP}_{i+1}-{AP}_{i}\right)}^{2}+{\left({ML}_{i+1}-{ML}_{i}\right)}^{2}}$$

where T = 30 s is the analyzed trial duration.

#### 2.5.3. Dynamic Postural Control

Dynamic balance was assessed using the SEBT (ICC ranged from 0.85 to 0.91) [36]. Participants stood on their affected limb, aligned at the intersection of the star grid, with their hands placed on the bilateral iliac crests to minimize upper body sway. The contralateral limb was used to reach as far as possible in three directions: anterior (A), posteromedial (PM), and posterolateral (PL), selected based on evidence that these directions are most sensitive to sensorimotor deficits in individuals with FAI [37]. The angle between A direction and both PL and PM directions was 135°, and the angle between PL and PM directions was 90°. A trial was deemed invalid and repeated if any of the following occurred: (1) the supporting foot was raised from the ground, (2) the reaching foot contacted the supporting surface, (3) weight was transferred to the reaching limb, or (4) the hands were removed from the iliac crests. Each participant completed three valid trials per direction. The maximum reach distance in each direction was marked by the examiner and measured accurately to 1 cm using a standard tape measure. All reach distances were normalized relative to leg length, measured as the straight-line distance from the anterior superior iliac spine to the medial malleolus.

Functional mobility was assessed using the SMHT. Participants were instructed to cover a 6 m distance as quickly as possible using consecutive single-leg hops on their affected limb, with self-selected hop length and pace. Prior to testing, standardized verbal instructions and a live demonstration were provided, followed by supervised practice trials to ensure task familiarization and minimize learning effects. Participants were encouraged to perform the test at a speed and intensity within their safe functional range, ensuring no risk of injury, and were informed they could terminate the test at any time if they felt unsafe. Timing was recorded using a digital stopwatch accurately to 0.01 s. A trial was deemed invalid and repeated if the non-stance limb contacted the ground or bore weight at any point during the task. The mean of three valid trials was used for analysis. This test has demonstrated good test–retest reliability in individuals with FAI (ICC = 0.80) [38].

### 2.6. Harms

Participants were explicitly instructed to report any discomfort, pain, skin irritation, or functional limitation related to device use during each 3-day telephone follow-up and at each assessment session. Additionally, at the end of the two-week intervention, all participants were asked questions regarding the presence of pain, localized pressure, itching, movement restriction, or any unintended musculoskeletal symptoms.

### 2.7. Procedure

Participants were assessed at three time points: pre-intervention, immediately post-intervention, and at the two weeks follow-up. Following eligibility screening and provision of written informed consent, participants attended three sessions.

During Session 1, demographic and clinical data were collected, and participants were randomly assigned to either the VAO or VFO groups. Anthropometric measurements were taken to enable device fit: ankle circumference (circumference measured just above the medial and lateral malleoli with the participant sitting and the ankle in a neutral 90° position) for VAO, and foot length, heel width, forefoot width, and medial arch height (measured with the participant standing in a relaxed bipedal stance to capture weight-bearing foot morphology) for VFO. Participants were scheduled to return for device fitting and baseline testing.

Session 2 was dedicated to baseline and immediate post-intervention assessments. Participants were fitted with their assigned intervention and received instructions on its fit and use. All participants used the interventions while wearing low-cut athletic shoes with flat, in-sole-free interiors. Athletic shoes typically provide a similar and sufficient sole rigidity, which helps standardize ankle-foot mechanics across participants. Baseline measurements (T1) were performed without the device. Immediately thereafter, participants wore the device for a 20 min adaptation period (walking with it), after which post-intervention assessments (T2) were conducted.

Following Session 2, participants were instructed to wear their assigned device for 4–8 h daily during weight-bearing activities, preferably outdoors, for two consecutive weeks. Those in both groups were asked to activate the vibrotactile system for 20 min, three times per week (Saturday, Monday, Wednesday), based on prior evidence indicating optimal neuromuscular adaptation within this dosing regimen and to avoid potential sensory receptor fatigue, habituation, or adverse effects from excessive stimulation [14]. Adherence was monitored every 3 days via telephone calls and short video submissions confirming device use. In Session 3, after two-week assessments (T3) were performed. All data (GRF data, reach distances, hop times, and mean absolute error) were collected by a single assessor.

### 2.8. Statistical Analysis

Sample size estimation was performed using G*Power (Version 3.1, University of Düsseldorf). Based on a prior study reporting a Cohen’s d effect size of 0.46 for COP velocity reduction with ankle orthosis [39], a statistical power of 0.60 (the minimum acceptable threshold for pilot trials), and a significance level of α = 0.10 (chosen to accommodate limited sample availability) [40], the target sample size was set at 8 participants per group.

Statistical analyses were performed using IBM SPSS Statistics software (version 26; IBM Corp., Armonk, NY, USA). No participant missed any assessment session. Group comparisons of baseline characteristics were performed using independent-samples *t*-tests for normally distributed continuous variables (verified with the Shapiro–Wilk test) and chi-square tests for categorical variables. Although a repeated-measures multivariate analysis of variance (MANOVA) was initially planned, high multicollinearity among the dependent variables, evidenced by variance inflation factors (VIF) > 5 and pairwise correlation coefficients > 0.5, necessitated the use of separate repeated-measures analyses of variance (ANOVAs) for each outcome. Each model examined the main effects of time, group, and the time × group interaction. At this stage, *p*-values < 0.05 were considered statistically significant.

When a significant interaction was observed, follow-up univariate repeated-measures comparisons were conducted to assess (1) between-group differences at each time point and (2) within-group changes across time points. If the interaction was non-significant, main effects were interpreted independently, with post hoc pairwise comparisons performed as appropriate. To control the Type I error rate across multiple outcomes, a Bonferroni correction was applied. With eight dependent variables, the adjusted alpha level was set at 0.006 (0.05 ÷ 8). Only *p*-values below this threshold were considered statistically significant in the post hoc tests.

Effect sizes were interpreted using established benchmarks: for repeated measures ANOVA, partial eta squared (η^2^) values of 0.01–0.06 indicated small effects, 0.06–0.14 medium effects, and >0.14 large effects. For post hoc pairwise comparisons, Cohen’s d < 0.2 denoted small effects, 0.5–0.8 medium effects, and >0.8 large effects.

## 3. Results

### 3.1. Participants Flow

**Figure 5:**
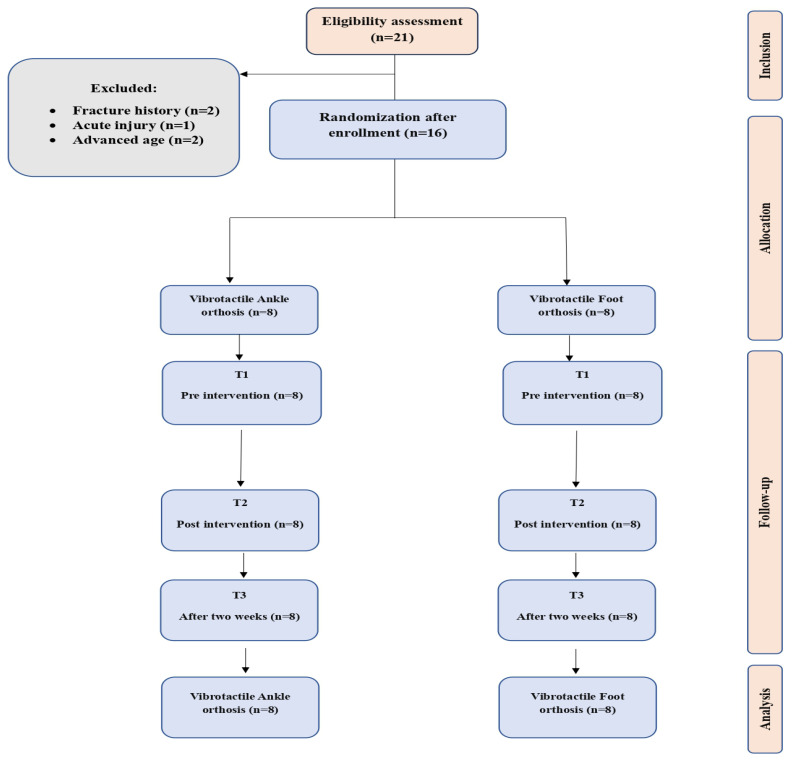
Flow diagram of participants.

Sixteen participants (n = 8 in each group) participated in this study. All participants completed all tests. No serious adverse events were reported. Two participants in the VAO group reported mild initial discomfort due to the bulk of the electronic housing, which resolved by the second week of use as they acclimatized to the device. All reported harms were documented, reviewed by the research team, and deemed non-serious. No participant withdrew from the study due to adverse effects. The flow diagram of this study is shown in Figure 5.

### 3.2. Baseline Data

The demographic and clinical characteristics of the participants at baseline are presented in Table 1. The two groups were comparable at baseline with respect to age, sex, body mass index, CAIT score, time since last LAS, and number of prior sprains (all *p* > 0.05).

### 3.3. Effects of Interventions

Analyses revealed a significant time × group interaction for JPS error (F (2,13) = 4.81, *p* = 0.02, partial η^2^ = 0.42), indicating differential effects between interventions. A significant main effect of time was observed for COP-AP velocity (F (2,13) = 23.66, *p* < 0.001, η^2^ = 0.78) and resultant COP velocity (F (2,13) = 6.93, *p* = 0.009, η^2^ = 0.51), reflecting overall improvement across the intervention period in both groups. No significant time × group interactions were found for COP-AP (*p* = 0.32), COP-ML (*p* = 0.66), or resultant COP velocity (*p* = 0.21). Similarly, no significant main effects of time, group, or interaction were observed for normalized SEBT reach distances in A, PM, and PL directions or SMHT completion time (*p* > 0.05 for all comparisons). Effect sizes for the significant outcomes were large, with partial eta squared values ranging from 0.42 to 0.78 (Table 2).

### 3.4. Change in Outcomes

Pairwise comparisons revealed that at the two-week follow-up (T3), the VFO group demonstrated significantly lower JPS error than the VAO group (mean difference = −1.75°, *p* = 0.005, Cohen’s d = −1.68), with no significant between-group differences at baseline (T1, *p* = 0.81) or immediately post-intervention (T2, *p* = 0.51). Within-group analyses showed that the VFO group exhibited significant improvements in JPS error between T1 and T3 (d = 1.64, *p* = 0.001) and T2 and T3 (d = 1.42, *p* = 0.004), whereas the VAO group showed negligible change across all time points (d ≤ 0.32, *p* ≥ 0.09).

Both groups demonstrated significant reductions in COP-AP velocity from T1 to T3 (VFO: d = 1.47, *p* = 0.003; VAO: d = 2.05, *p* < 0.001). The resultant COP was significantly decreased in the VAO group at T1 and T2 (d = 1.38, *p* = 0.005). No other pairwise comparisons for COP mediolateral, resultant velocity, SEBT, or SMHT reached statistical significance (*p* > 0.05) (Table 3).

**Figure 6:**
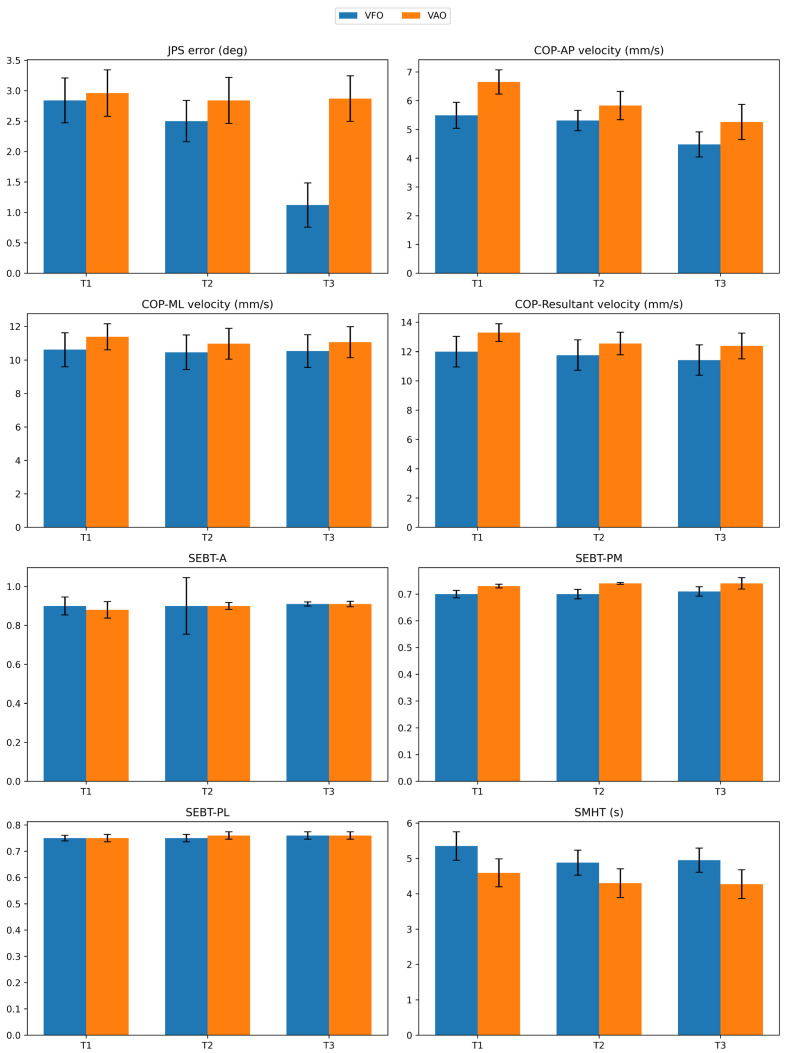
Bar charts showing the mean (±SEM) values of all outcome measures at baseline (T1), immediately post-intervention (T2), and at the two-week follow-up (T3) for the VFO and VAO groups.

Figure 6 displays clustered bar charts of the mean (±SEM) values for all outcome measures at baseline (T1), immediately post-intervention (T2), and two-week follow-up (T3), sepa-rately for the VFO and VAO groups.

## 4. Discussion

This pilot trial is the first to investigate the efficacy of ankle- versus plantar-based vibrotactile orthoses on JPS, static postural stability, and dynamic balance in young adults with FAI. The VFO demonstrated superior effectiveness compared to the VAO in improving JPS at the two-week follow-up. Regarding postural stability, the two groups showed a significant reduction in COP-AP velocity after two weeks. Additionally, the VAO group demonstrated a significant decrease in COP-R velocity at immediate assessment. These improvements align with previous research suggesting that perceptible vibratory stimulation enhances intrinsic proprioceptive feedback [41,42,43,44,45,46,47,48]. Despite the small sample size, large effect sizes were observed for both interventions on significant outcomes, suggesting clinically meaningful changes. Furthermore, participants responded positively to the devices.

Studies investigating vibratory orthoses have focused on their effects on postural stability in individuals with diabetic neuropathy [41,42], Parkinson’s disease [43], knee osteoarthritis [44], stroke [45], cerebral palsy [46], older adults [47], and healthy individuals [48]. Results consistently show reductions in COP sway velocity [40,41,43,46,47], elliptical area [42,43], and root mean square [48], adduction moment [44], which can be attributed to the biomechanical support and proprioceptive enhancement provided by the devices. Currently, orthoses available to young adults with FAI for improving balance are limited to passive ankle supports, insoles, and high-top shoes [49]. Although substantial evidence supports the use of focal vibrotactile stimulation in the foot and ankle region to improve proprioception [29], balance, and gait in young adults [48], little to no research has examined the efficacy of integrating this stimulus into orthoses specifically designed for the FAI biomechanics.

The VFO improved JPS compared to the VAO device at the two-week follow-up. These results imply that mechanoreceptors in the ankle ligaments play a lesser role in ankle proprioception compared to those located in the plantar region of the foot [29,50,51,52]. Tactile input from cutaneous mechanoreceptors in the plantar surface of the feet conveys information to the CNS regarding the distribution of pressure under the feet [6]. Alterations in this pressure pattern are frequently associated with shifts in upright postural alignment and spatial position sense [53]. Additionally, the differential effects observed between VFO and VAO may be explained by the distinct stimulation demands of the injured ankle region versus the plantar surface. In individuals with FAI, mechanoreceptors around the lateral ankle ligaments are particularly susceptible to injury, reducing localized afferent input [8]. Vibrotactile stimulation at the ankle may therefore target deficits related to ligamentous damage [16]. Conversely, the plantar surface of the foot contains a high density of mechanoreceptors that continuously interact with ground reaction forces, providing rich proprioceptive information for balance and postural control [15,16]. Stimulation at this site may enhance the integration of tactile and proprioceptive cues at the foot-ground interface, thereby producing greater improvements in JPS.

Both interventions decreased postural sway during use. But there was no between-group significance in this parameter immediately or after two weeks. In the past studies, the effect of AOs and FOs on postural sway was quantified using COP parameters, including mean displacement, root mean square, COP slope, acceleration, and velocity, all assessed in both the AP and ML directions [20,21,22]. Among these metrics, COP velocity was selected as the primary outcome due to its high reliability, and sensitivity to sensorimotor deficits in individuals with FAI [34]. It is posited that orthotic mechanisms that are capable of addressing subtalar axis deviations or enhancing stability of the talocrural joint could offer reductions in postural sway without between-group superiority [25]. The lateral shift in plantar pressure toward the lateral aspect of the foot compromises the ankle’s capacity to effectively modulate postural sway, thereby increasing reliance on proximal hip-based control mechanisms [9]. Consequently, the functional role of ankle musculature becomes critical not only for fine-tuning postural adjustments but also for coordinating complex sensorimotor tasks during dynamic activities [8]. It seems that enhanced both mechanical support and tactile stimuli can reweight the sensorimotor mechanism of balance that can be quantified through COP sway. GRF varies significantly with activity level, being relatively low during quiet standing and higher during dynamic tasks such as running or jumping [25]. Accordingly, the present study tested a low-load condition, and future investigations are warranted to examine whether similar benefits of vibrotactile orthoses are observed under high-impact activities commonly associated with ankle instability.

Although the improvements in COP mediolateral velocity, SEBT scores, and SMHT time did not reach statistical significance, the observed effect sizes ranged from small to large. A post hoc power analysis, based on the observed effect sizes and pooled within-group variability, indicates that a sample size of 10 to 23 participants per group would provide 80% power (α = 0.05, two-tailed) to detect these effects as statistically significant in a future definitive trial. Critically, both intervention groups exhibited consistent directional improvements: SEBT reach distances increased across all three directions (particularly PM), and hop times decreased by 0.3–0.5 s, changes that exceed the minimal clinically important difference (MCID) reported for these measures in FAI [54,55]. These consistent trends, combined with large effect sizes, suggest that the non-significant findings are attributable to limited statistical power rather than a true absence of effect.

The effectiveness of VFO in enhancing JPS indicates that clinicians should focus on improving sensory feedback at the foot-ground interface when treating individuals with FAI. Nevertheless, it is crucial to consider patient classifications: those with additional ligamentous laxity or mechanical instability may benefit from using VAO, which offers both mechanical support and sensory stimulation. Incorporating these devices into rehabilitation protocols requires a structured approach. During the acute recovery phase after recurrent sprains, passive ankle orthoses are the primary intervention [3,25]. The use of VFO is most suitable during the subacute phase (4–8 weeks post-injury), which focuses on sensorimotor retraining. We recommend starting with daily 20 min sessions of VFO during weight-bearing activities, gradually advancing to dynamic balance exercises as JPS improves. The VAO can be introduced later in the rehabilitation process when controlled perturbation training begins, utilizing its mechanical stability while also benefiting from vibration-induced neuromuscular facilitation [56].

This study has several limitations. Firstly, this study’s small sample size limits statistical power, although appropriate for an efficacy pilot trial. Secondly, the intervention duration was brief (two weeks), which may be insufficient to induce meaningful neuroplastic adaptations in dynamic balance or functional mobility. Furthermore, the absence of a post-removal follow-up assessment prevents distinguishing between temporary assistive effects and genuine, retained neuroplastic learning. Future studies should therefore adopt longer intervention periods and include follow-up assessments to clarify the durability of observed improvements. Our study did not measure or control for potential confounding factors such as plantar pressure distribution assessed by foot scanners, prior rehabilitation history, and daily physical activity levels. These factors may have influenced proprioceptive outcomes and could partly explain variability in the VFO versus VAO comparison on joint position sense. Another limitation of the present study is that the vibrotactile stimulus was treated as a simple on/off signal, without controlling for or measuring its dynamic properties at the anatomical target (e.g., damping, vibration transmission through tissue, local resonant frequencies). Other studies should incorporate vibration engineering analyses to characterize these properties, thereby improving reproducibility and advancing understanding of the mechanisms underlying vibrotactile stimulation. Finally, the sample consisted exclusively of young adults with unilateral FAI, limiting generalizability to older populations, adolescents, individuals with bilateral instability, or those with comorbidities such as diabetic neuropathy or osteoarthritis, conditions that may alter mechanoreceptor density and central sensorimotor integration. Future studies should explore dose–response relationships by systematically varying vibrotactile parameters, such as frequency, amplitude, and duty cycle, to identify optimal stimulation protocols that maximize neuromodulatory and sensorimotor benefits. Additionally, integrating neurophysiological measures (e.g., surface electromyography and cortical evoked potentials) would help elucidate the central and peripheral mechanisms driving the observed improvements in proprioception and postural control. Finally, to enhance ecological validity, real-world adherence and functional carryover should be assessed using wearable inertial sensors during activities of daily living, providing objective data on how laboratory-based gains translate into meaningful improvements in everyday mobility and stability.

## 5. Conclusions

This pilot randomized trial demonstrates that plantar-based vibrotactile orthosis (VFO) is superior to ankle-based stimulation (VAO) for enhancing JPS in individuals with FAI, with its effects manifesting after two weeks of usage. Static postural control was significantly enhanced by both interventions, as evidenced by reduced COP velocity in the anteroposterior direction, without between-group differences for this measure. The findings suggest that plantar cutaneous input is more significant than ankle mechanoreceptors in re-establishing proprioceptive acuity in this cohort. Despite the small sample and short intervention duration, the effect sizes demonstrate clinically relevant change.

## Figures and Tables

**Table 1:** The characteristics of participants.

Characteristics	VAO M ± SD or No.	VFO M ± SD or No.
Age (years)	22.6 ± 6.3	23.8 ± 5.8
Sex (male/female)	3/5	4/4
Body mass index (kg/m^2^)	24.6 ± 4.2	23.1 ± 5.8
CAIT (0–30)	19.3 ± 3.1	20.3 ± 1.9
Time since last ankle sprain (months)	4.3 ± 7.9	5.8 ± 6.32
Number of previous ankle sprains	4.09 ± 5.02	5.52 ± 3.26
Dominant side (right/left)	7/1	5/3
Affected side (right/left)	6/2	6/2

M: Mean, SD: Standard Deviation.

**Table 2:** Descriptive statistics and results of repeated measures ANOVA.

Outcome	Groups	Time			Group × Time	Time	Group
		T1 (M ± SD)	T2 (M ± SD)	T3 (M ± SD)			
Error of ankle JPS (Degrees)	VFO	2.84 ± 1.04	2.5 ± 0.96	1.12 ± 1.03	Wilk’s Lambda = 0.57 F (2,13) = 4.81 *p* = 0.02 * η^2^ = 0.42	Wilk’s Lambda = 0.54 F (2,13) = 5.53 *p* = 0.01 * η^2^ = 0.46	F (2,13) = 2.74 *p* = 0.12 η^2^ = 0.16
	VAO	2.96 ± 1.08	2.84 ± 1.07	2.87 ± 1.06			
Mean velocity of COP-AP (mm/s)	VFO	5.49 ± 1.28	5.31 ± 1	4.48 ± 1.23	Wilk’s Lambda = 0.84 F (2,13) = 1.24 *p* = 0.32 η^2^ = 0.16	Wilk’s Lambda = 0.21 F (2,13) = 23.66 *p* ˂ 0.001 * η^2^ = 0.78	F (2,13) = 1.68 *p* = 0.21 η^2^ = 0.10
	VAO	6.65 ± 1.19	5.83 ± 1.39	5.26 ± 1.73			
Mean velocity of COP-ML (mm/s)	VFO	10.61 ± 2.86	10.46 ± 2.91	10.53 ± 2.74	Wilk’s Lambda = 0.93 F (2,13) = 0.42 *p* = 0.66 η^2^ = 0.06	Wilk’s Lambda = 0.76 F (2,13) = 2.05 *p* = 0.16 η^2^ = 0.24	F (2,13) = 0.2 *p* = 0.65 η^2^ = 0.01
	VAO	11.38 ± 2.21	10.97 ± 2.59	11.06 ± 2.61			
Mean velocity of COP-Resultant (mm/s)	VFO	11.99 ± 2.94	11.76 ±2.93	11.42 ± 2.92	Wilk’s Lambda = 0.78 F (2,13) = 1.73 *p* = 0.21 η^2^ = 0.21	Wilk’s Lambda = 0.48 F (2,13) = 6.93 *p* = 0.009 * η^2^ = 0.51	F (2,13) = 0.64 *p* = 0.43 η^2^ = 0.04
	VAO	13.29 ± 1.71	12.55 ± 2.16	12.38 ± 2.47			
SEBT-A (normalized to leg length)	VFO	0.9 ± 0.13	0.9 ± 0.41	0.91 ± 0.03	Wilk’s Lambda= 0.9 F (2,13) = 0.06 *p*= 0.93 η^2^ = 0.01	Wilk’s Lambda = 0.9 F (2,13) = 0.69 *p* = 0.51 η^2^ = 0.09	F (2,13) = 0.31 *p* = 0.58 η^2^ = 0.02
	VAO	0.88 ± 0.12	0.9 ± 0.05	0.91 ± 0.04			
SEBT-PM (normalized to leg length)	VFO	0.7 ± 0.04	0.7 ± 0.05	0.71 ± 0.05	Wilk’s Lambda = 0.95 F (2,13) = 0.29 *p* = 0.74 η^2^ = 0.04	Wilk’s Lambda= 0.97 F (2,13) = 0.18 *p* = 0.83 η^2^ = 0.02	F (2,13) = 3.84 *p* = 0.07 η^2^ = 0.21
	VAO	0.73 ± 0.02	0.74 ±0.01	0.74 ± 0.06			
SEBT-PL (normalized to leg length)	VFO	0.75 ± 0.03	0.75 ± 0.04	0.76 ± 0.04	Wilk’s Lambda= 0.85 F (2,13) = 1.06 *p* = 0.37 η^2^ = 0.14	Wilk’s Lambda= 0.75 F (2,13) = 2.1 *p* = 0.16 η^2^ = 0.14	F (2,13) = 0.01 *p* = 0.92 η^2^ = 0.001
	VAO	0.75 ± 0.04	0.76 ± 0.04	0.76 ± 0.04			
SMHT (s)	VFO	5.35 ± 1.14	4.88 ± 1	4.95 ± 0.97	Wilk’s Lambda= 0.95 F (2,13) = 0.3 *p* = 0.75 η^2^ = 0.04	Wilk’s Lambda= 0.72 F (2,13) = 2.50 *p* = 0.12 η^2^ = 0.27	F (2,13) = 1.69 *p* = 0.21 η^2^ = 0.1
	VAO	4.59 ± 1.12	4.30 ± 1.15	4.27 ± 1.15			

* *p* < 0.05 was considered statistically significant.

**Table 3:** Pairwise comparison.

Pairwise Between-Group Comparisons at Each Time Point				
Outcome	Time	Conditions	MD ± SE (95%CI)	*p* Value (Cohen’s d)
Error of ankle JPS (Degrees)	T1	VFO vs. VAO	−0.12 ± 0.53	0.81 (−0.11)
			(−1.26 to 1.01)	
	T2	VFO vs. VAO	−0.34 ± 0.51	0.51 (−0.33)
			(−1.44 to 0.75)	
	T3	VFO vs. VAO	−1.75 ± 0.52	0.005 * (−1.68)
			(−2.87 to −0.62)	
Pairwise comparisons of time points within each group				
Outcome	Group	Conditions	MD ± SE (95%CI)	*p* value (Cohen’s d)
Error of ankle JPS (Degrees)	VFO	T1 vs. T2	0.34 ± 0.27	0.68 (0.45)
			(−0.39 to 1.08)	
		T1 vs. T3	1.71 ± 0.37	0.001 * (1.64)
			(0.7 to 2.73)	
		T2 vs. T3	1.37 ± 0.34	0.004 * (1.42)
			(0.43 to 2.31)	
	VAO	T1 vs. T2	0.12 ± 0.27	1 (0.16)
			(−0.61 to 0.86)	
		T1 vs. T3	0.09 ± 0.37	1 (0.09)
			(−0.61 to 0.86)	
		T2 vs. T3	−0.31 ± 0.34	1 (−0.32)
			(−0.97 to 0.91)	
Mean velocity of COP-AP (mm/s)	VFO	T1 vs. T2	0.18 ± 0.28	1 (0.23)
			(−0.58 to 0.94)	
		T1 vs. T3	1 ± 0.24	0.003 * (1.47)
			(0.33 to 1.67)	
		T2 vs. T3	0.82 ± 0.23	0.01 (1.26)
			(0.17 to 1.47)	
	VAO	T1 vs. T2	0.82 ± 0.28	0.03 (1.03)
			(0.06 to 1.58)	
		T1 vs. T3	1.39 ± 0.24	˂0.001 * (2.05)
			(0.72 to 2.06)	
		T2 vs. T3	0.56 ± 0.23	0.09 (0.86)
			(−0.08 to 1.21)	
Mean velocity of COP-R (mm/s)	VFO	T1 vs. T2	0.23 ± 0.19	0.72 (0.43)
			(−0.28 to 0.75)	
		T1 vs. T3	0.57 ± 0.32	0.3 (0.63)
			(−0.31 to 1.46)	
		T2 vs. T3	0.34 ± 0.26	0.65 (0.46)
			(−1.06 to 0.37)	
	VAO	T1 vs. T2	0.74 ± 0.19	0.005 * (1.38)
			(0.22 to 1.25)	
		T1 vs. T3	0.91 ± 0.32	0.04 (1.01)
			(0.03 to 1.08)	
		T2 vs. T3	0.17 ± 0.26	1 (0.23)
			(−0.54 to 0.89)	

* *p* < 0.006 was considered statistically significant.

## Data Availability

The data supporting the findings of this study are available from the corresponding authors upon reasonable request. Due to privacy and ethical restrictions, the data are not publicly deposited.

## Abbreviations

The following abbreviations are used in this manuscript:

LAS	Lateral ankle sprain
FAI	Functional ankle instability
JPS	Joint position sense
GRF	Ground reaction force
CNS	Central nervous system
FOs	Foot orthoses
AOs	Ankle orthoses
VAO	vibrotactile ankle orthoses
VFO	vibrotactile foot orthoses
PWM	Pulse width modulation
EVA	Ethylene-vinyl acetate
SEBT	Star Excursion Balance Test
SMHT	Six-Meter Hop Test
A	Anterior
PM	Posteromedial
PL	Posterolateral
AP	Anteroposterior
ML	Mediolateral
η^2^	partial eta squared
CAIT	Cumberland ankle instability tool
COP-R	Center of pressure resultant
MCID	minimal clinically important difference
